# Delivery of AntagomiR-7 through polymer nanoparticles for assisting B Cell to alleviate systemic lupus erythematosus

**DOI:** 10.3389/fbioe.2023.1180302

**Published:** 2023-04-19

**Authors:** Hui Guo, Jiangtao Ma, Yanli Zhang, Yan Mao, Ziwei Hu, Ying Lin, Feng Yu, Wei Wang, Yaling Liu

**Affiliations:** ^1^ Department of Dermatology, The Third Hospital of Hebei Medical University, Shijiazhuang, HB, China; ^2^ Hebei Orthopedic Clinical Research Center, Orthopaedic Institution of Hebei Province, The Third Hospital of Hebei Medical University, Shijiazhuang, HB, China; ^3^ Institute of Otolaryngology, Head and Neck Surgery, Guangzhou Red Cross Hospital of Jinan University, Guangzhou, GD, China; ^4^ School of Pharmaceutical Sciences, Southern Medical University, Guangzhou, GD, China

**Keywords:** antagomiR-7, SA-PLGA@antagomiR-7, nanoparticle, B cell hyperresponsiveness, systemic lupus erythematosus

## Abstract

An autoimmune condition known as systemic lupus erythematosus (SLE) is characterized by B cell hyperresponsiveness and persistent generation of pathogenic autoantibodies that cause damage to various organs and tissues. The treatments available today are either ineffective or have adverse effects. The dysregulation of B cell activation is crucial for the emergence of SLE. MiR-7 explicitly targeted PTEN mRNA in B cells. Treatment with antagomiR-7 reduced B cell hyperresponsiveness and prevented the onset of lupus. As a result, inhibiting miR-7 may be used therapeutically to treat SLE. We developed a SA (sialic acid)-poly (D, L-lactide-co-glycolide) (SA-PLGA) nano delivery system to deliver antagomiR-7 into splenic B cells since the stability and targeted delivery of miRNA remain significant challenges *in vivo*. Results show that SA-PLGA nanoparticles (SA-PLGA@antagomiR-7) loaded with antagomiR-7 display good biocompatibility and shield antagomiR-7 from degradation, extending the miRNA’s duration in circulation *in vivo*. Additionally, in MRL/Ipr lupus mice, SA-PLGA@antagomiR-7 is successfully delivered to the splenic B cells and preferentially enriched in the diseased spleen in MRL/Ipr lupus mice. The SA-PLGA@antagomiR-7 NPs therapy effectively decreases immunological abnormalities, normalizes splenic B cell subtypes, and suppresses B cell activation. The antagomiR-7 NPs exhibit excellent therapeutic efficiency and high biosafety collectively, which may result in a more effective treatment for SLE.

## 1 Introduction

Systemic lupus erythematosus (SLE) is a multisystem autoimmune disease characterized by loss of tolerance and continuous production of autoantibodies against autoantigens that form deposits of immune complexes (Lisnevskaia et al., 2014). The reported prevalence of SLE worldwide is 20–150 cases per 1,00,000 people and a male-to-female ratio of ∼1:9 (Durcan et al., 2019). Most scholars now believe that a combination of genetic susceptibility and environmental, immunological, and hormonal factors contribute to SLE, but the exact mechanism is unknown (Vyse and Kotzin, 1998; Tsokos et al., 2016; Dörner and Furie, 2019). Glucocorticoids (GCs), immunosuppressive agents and biologics are currently commonly used to treat SLE (Gatto et al., 2019; Fanouriakis et al., 2021). However, the efficacy of such therapies is not satisfactory and cannot provide adequate control of SLE. In addition, these drugs suffer from toxic side effects, lack of target tissues, and therapeutic unresponsiveness, among other barriers (Trentin et al., 2018; Parra Sánchez et al., 2022). Therefore, exploring highly effective and safe treatments for SLE is necessary.

The key to the pathogenesis of SLE is B cell hyperresponsiveness and pathogenic autoantibody production, which contribute to the deposition of immune complexes in multiple organs, chronic inflammation, and organ damage (Ceccarelli et al., 2015). Many studies have shown miRNAs’ potential as disease biomarkers, identifying specific dysregulated pathways involved in disease pathogenesis and possible therapeutic targets (Chen et al., 2016; Lu et al., 2019). Previous studies found that in human SLE, miR-7 inhibitors directly targeted the PTEN 3′-UTR, inhibited PTEN mRNA and protein expression, and inappropriate activation of the AKT signalling pathway in lupus B cells (Wu et al., 2014). Elevated miR-7 levels were positively correlated with reduced PTEN expression, a high percentage of plasma cells and increased pAKT in B cells (Wang et al., 2020). Furthermore, blocking miR-7 with antagomiR-7 corrected the increased differentiation of B cells to plasma cells, a biological process mediated by regulation of the PTEN/AKT signalling pathway, while inhibiting disease activity in lupus (Wang et al., 2020). MiRNA antagomir is a chemically modified miRNA antagonist that inhibits miRNA action by binding strongly and competitively to mature miRNAs *in vivo*, preventing complementary pairing of miRNAs with their target gene mRNAs. AntagomiR-7 is a promising target for treating SLE in regulating PTEN expression and B cell activation (Wang et al., 2020).

Sialic acid oligosaccharides found at the cell membrane interface are involved in a wide range of biological processes, such as cell-cell interactions and aspects of glycoimmunology (Varki and Gagneux, 2012). Many pathogens decorate their surfaces with specific sialic acid derivatives to immobilize and infect specific cells (Stafford et al., 2012; Liu et al., 2018; Kong et al., 2019). CD22 is a cell surface receptor expressed exclusively on B cells, which is involved in the B cell receptor (BCR) signalling pathway (Nitschke, 2009). CD22 is essential in B cell activation and differentiation and is an attractive target for B cell-specific drug delivery (O'Reilly and Paulson, 2009). The role of CD22 depends on extracellular interactions with ligands carrying sialic acid. This mechanism is essential to ensure humoral responses but also prevents unwanted autoimmune responses to self-antigens and prevents autoimmunity (St-Pierre et al., 2016). Therefore, targeting CD22 is of interest to SLE. N-acetylneuraminic acid (Neu5Ac), also known as sialic acid, is known for its involvement in cellular recognition and can act as a ligand to deliver drugs to CD22 (Dörner et al., 2015; Mi et al., 2021).

With recent advances in nanotechnology, nanoparticle (NP)-based diagnostics and therapeutics have shown considerable success in clinical trials (Kapadia et al., 2020; Li et al., 2021; Li H. et al., 2022; Wang et al., 2022). Nanoparticles have several advantages, including improved pharmacokinetic profiles of cytotoxic drugs, targeting capabilities and reduction of their toxicity (Ban et al., 2019; Liao et al., 2020; Wang et al., 2021; Li et al., 2023). Notably, several nanoparticle-based drug delivery systems have been reported to reduce the progression of SLE in mouse models effectively. For example, phosphatidylserine (PS) surface-modified gold nanocages loaded with LXR agonists inhibit lupus manifestations in mice by targeting PS recognition receptors specific to the surface of macrophages, promoting phagocytic receptor expression and enhancing their ability to phagocytose apoptotic cells (Xu et al., 2019). Biodegradable spherical capsules composed of polypropylene glycol (PLA)/polyethene glycol (PEG) polymers encapsulated CsA, targeting CD71 and thus enhancing lymphoid tissue targeting, reduced the dose of CsA used (Ganugula et al., 2020). For bimodal imaging and therapy of lupus nephritis, polydopamine (PDA)-based nanocarriers were modified with Fe_3_O_4_ and Pt nanoparticles (PDA@Pt-Fe_3_O_4_) and loaded with the RIPK1 inhibitor Nec-1 (Li M. et al., 2022). By stimulating Tregs, IL-2-loaded PLGA nanoparticles that target T cells prevent BDF1 mice from developing lupus-like disease (Ferretti et al., 2021). Nanoparticles (IM-MNPs/DXM) were prepared from cancer cell antigen-presenting molecule (MHC-I)-deficient cell membranes loaded with dexamethasone (DXM). IM-MNPs acquire cell membrane properties that enable them to accumulate in inflamed organs and avoid immune clearance. They also target CD4^+^ T cells and agonize PD-1/TIGIT signaling to impair effector T cell function (Guo et al., 2022). In addition, prior studies constructed miR-125a-loaded nano-delivery systems to reverse the effector/regulatory T-cell imbalance to significantly mitigate SLE disease progression (Pan et al., 2015; Zhang et al., 2020). These studies suggested that using nanomedicine to treat autoimmune diseases is a promising approach. A characteristic of SLE is aberrant B cell overactivation, which is essential for the onset and chronic duration of the illness. However, as far as we know, no reported studies are using nano-delivery systems to target B cells for miRNA-based SLE gene therapy. Herein, we explored NPs targeting B-cell gene therapy for SLE.

**SCHEME 1:**
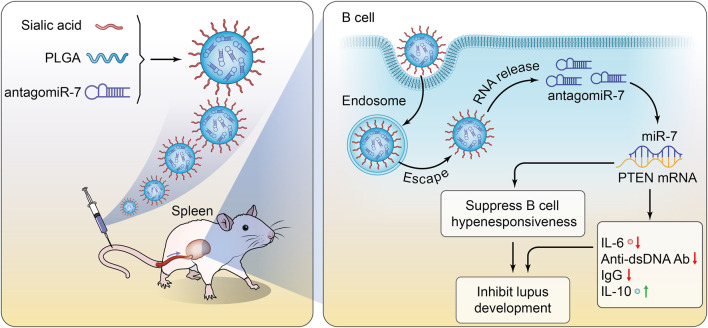
Schematic illustration of SA-PLGA@antagomiR-7 inhibits B cell overresponse to alleviate systemic lupus erythematosus.

In this study, we successfully prepared a PLGA@ antagomiR-7 nano-delivery system for B cell gene therapy by conjugating sialic acid (SA) on the surface of the PLGA NPs for antagomiR-7 delivery. The PLGA NPs were decorated with sialic acid to allow selective recognition of CD22 on B cell surfaces. SA constitutes a characteristic feature of NPs and could recognize signals by binding to the CD22 expressed on B cells. SA-PLGA@ antagomiR-7 could significantly increase the PTEN expression on B cells. In addition, SA-PLGA@ antagomiR-7 shows excellent safety, decreased anti-dsDNA autoantibodies production, and reduced systemic inflammation and kidney damage. Overall, the SA-PLGA NPs provide a promising strategy for SLE therapy (Scheme 1).

## 2 Materials and methods

### 2.1 Preparation of SA-PLGA@antagomiR-7

To 1 mL of chloroform, mix 20 mg of poly (d, l-lactide-coglycolide) (50:50 PLGA, Aladdin, China). The PLGA solution was then mixed with 5 nmol of antagomiR-7 (Ruibo Biotechnology, China) dissolved in 5 mL of DEPC water, followed by 1 min of contact in an ice bath sonicator (100 W). After sonicating once more for 10 min, 1 mg of N-acetylneuraminic acid (Yuanye Biotechnology, China) that had been dissolved in DEPC water was added. Chloroform was then eliminated through room-temperature evaporation. The nanoparticles’ hydrodynamic size, surface zeta potential and polydispersity index (PDI) were then assessed with a Zeta-sizer (Malvern, UK). Over 7 days, the nanoparticle stability in 1 × PBS was investigated. Transmission electron microscopy (TEM, JEM-1400 PLUS 120 kV) was used to validate and visualize the nanoparticles’ existence.

### 2.2 miRNA protection and release of SA-PLGA@antagomiR-7

SA-PLGA@antagomiR-7 nanoparticles were treated with RNAase A at 37°C for 2h, 6h, 12h, and 24 h to examine the protective impact of antagomiR-7. Then, a 5% agarose gel containing 10 μL of nucleic acid dye was electrophoresed at 90 V for 20 min. A GelDoc XR imaging device was then used to see the gels (Bio-Rad, United States). As a reference, free antagomiR-7 (100 nmol) was employed.

Analyses of the miRNA release from SA-PLGA@antagomiR-7 NP were done using dialysis assays. A dialysis bag contained 2 mL of SA-PLGA@antagomiR-7 solution that had been tagged with Rho. After that, 18 mL of DEPC water was added to the dialysis bag and gently shaken at 37°C. At a predetermined time, removed 200 μL of the external solution and added the same medium volume to the external solution to measure the Rho NP fluorescence intensity using a multifunctional enzyme marker (PerkinElmer, Victor Nivo, Finland). The cumulative release rate (%) = (cumulative release of Rho NP/total quantity of Rho NP in the feed) 100% was used to calculate the cumulative release of Rho from RhB-SA-PLGA@antagomiR-7 NP.

### 2.3 Preparation and culture of primary splenocytes

Splenocytes were prepared by lightly disrupting 70 μm cell filters of the spleen. Erythrocytes were lysed with RBC lysis buffer (Solarbio, China). The splenocytes were subjected to magnetic bead sorting, using CD19 antibody-biotin (Miltenyi Biotec, Germany) and Anti-biotin microbeads (Miltenyi Biotec, Germany) according to the manufacturer’s recommendations, to obtain purified splenic B cells. Cells were then cultured in 1,640 medium containing 10% FBS, anti-IgM (10 μgmL^−1^, Thermo, United States), CD40L (100 ng mL^−1^, RD, United States) and IL-4 (20 ng mL^−1^, RD, United States), 100 U/ml penicillin and streptomycin in 5% CO_2_.

### 2.4 Cytotoxicity assay and cellular uptake

Splenocytes were treated with different concentrations of Free antagomiR-7, PLGA@antagomiR-7 and SA-PLGA@antagomiR-7 (100, 200, 400 and 800 μg/mL) for 24 h. Afterwards, cell viability was assessed by a CCK8 kit (Dojindo, Japan), and cell viability was measured by multifunctional enzyme labelling. Cell viability was assessed by CCK8 kit, and optical density was measured at 450 nm using a multifunctional enzyme marker. The mean fluorescence intensity of B cell phagocytosis in lupus mice was determined by flow cytometric analysis after 4 h co-culture with Rho-PLGA@antagomiR-7 or Rho-SA-PLGA@antagomiR-7.

### 2.5 Flow cytometry analysis

Splenic lymphocytes were stained with various combinations of monoclonal antibodies (mAb) against surface markers in a staining buffer for 30 min on ice. The following directly conjugated monoclonal antibodies were used: anti-CD19-FITC (BD biosciences, 1D3, United States), anti-CD21-Percp-Cy5.5 (BD biosciences, 7E9, United States), anti-CD23-PE (BD biosciences, B3B4, United States), anti-CD138-APC (BD biosciences, 281–2, United States). Stained cells were detected by FACS flow cytometry (BD FACSCelesta, United States). Data were analyzed with FlowJo software.

### 2.6 Quantification of mRNA and miRNA

Harvest freshly isolated or cultured purified B cells. Total RNA was extracted from the cell samples using an RNA Easy Fast Tissue/Cell kit (Tiangen, China) and reversed transcribed to cDNA using the miRcute Plus miRNA First-Strand cDNA Kit (Tiangen, China) according to the manufacturer’s protocol. miR-7 TaqMan probe analysis was performed in 7,500 Real-Time PCR (ThermoFisher) using the miRcute Plus miRNA qPCR Kit (Tiangen, China). Expression levels were standardized relative to U6. Total RNA was extracted using the RNeasy Mini Kit (Qiagen, China). Reverse transcription reactions were prepared using the PrimeScript RT reagent kit with gDNA Eraser (Takara, Japan). Real-Time PCR was performed using TB Green Premix TaqⅡ (Takara, Japan). The expression level was standardized as GAPDH. The relative expression of mRNA and miRNA was analyzed by relative quantification (2^−ΔΔCT^). All values are expressed as mean ± standard deviation. Statistical analysis was performed using GraphPad Prism 8 software.

### 2.7 Western blotting

Purified spleen B cells were isolated by negative selection using CD19 microbeads according to the manufacturer’s recommendations. Cells were lysed in RIPA buffer in a mixture of protease and phosphatase inhibitors for protein extraction. Protein concentrations were determined by using the BCA Protein Assay Kit (Beyotime, China). Equal amounts of proteins were resolved by SDS-PAGE, transferred to PVDF membranes, and diluted with p-mouse PTEN (1:1,000 dilution; Cell Signaling Technology, United States). Corresponding horseradish peroxidase (HRP)-conjugated secondary antibodies are used for visualization by enhanced chemiluminescence (ECL) reactions.

### 2.8 Biodistribution of NPs *in vivo*

C57BL/6 female mice (Hfkbio Laboratory Animal Co. LTD.) were injected intravenously with DIR-SA-PLGA@antagomiR-7 or DIR-PLGA@antagomiR-7 at a dose of 1.5 mg/kg. *In vivo* fluorescence was measured using a fluorescence imaging system (Ivis Spectrum CT, United States) at 4 h, 12 h, and 24 h post-injection, and then fluorescence images were captured.

### 2.9 *In vivo* application

Randomly assigned 10-week-old MRL/Ipr female mice (Cavens Laboratory Animal Co. LTD., China): divided into four groups of PBS, Free antagomiR-7, PLGA@antagomiR-7 and SA-PLGA@antagomiR-7. Mice (antagomiR-7 at a dose of 1.5 mg/kg) were injected intravenously twice a week (weeks 10, 11, 12 and 13). All animal experiments were carried out according to protocols approved by the Experimental Animal Ethics Committee of Hebei Ex&lnvivo Biotechnology Co., Ltd. Urine was collected weekly to measure proteinuria by the BCA protein assay kit (Beyotime, China). Three days after the last treatment, the mice were then euthanized, and blood was collected. The spleen and axillary lymph nodes were weighed. The kidneys were fixed, embedded in paraffin, and then processed by HE staining analysis. Serum creatinine (CRE) and urea nitrogen (BUN) were measured by a fully automated biochemical analyzer (Rayto Chemray 240, China). All animal experiments were conducted using methods approved by Hebei Ex&lnvivo Biotechnology Co., Ltd.’s Experimental Animal Ethics Committee.

### 2.10 ELISA

Serum concentrations of IL-6 (RD, United States), IL10 (Mlbio, Shanghai, China), Anti-dsDNA Ab (Mlbio, Shanghai, China) and IgG (Mlbio, Shanghai, China) were assessed by ELISA. Absorbance was measured using a multifunctional enzyme marker following the guidelines of the various protocols.

### 2.11 Histopathological evaluation of the kidney

Paraffin sections (5 μm) were deparaffinized and stained with hematoxylin and eosin (H&E). These sections were then dehydrated with gradient ethanol and transparent with xylene. H&E sections were viewed by light microscopy. Two nephrologists evaluated and scored glomerulonephritis on a blinded scale of 0–4: 0, normal; 1, 1%–25% injury; 2, 26%–50% injury; 3, 51%–75% injury; 4, > 75% injury.

### 2.12 Safety assessment

Randomly assigned 10-week-old C57 female mice (Hfkbio Laboratory Animal Co. LTD.): PBS, Free antagomiR-7, PLGA@antagomiR-7, SA-PLGA@antagomiR-7 and cyclophosphamide groups. Mice (antagomiR-7 at a dose of 1.5 mg/kg) were injected intravenously twice a week (weeks 10, 11, 12, 13) twice a week. Treatment for 1 month. Blood was collected 3 days after the last treatment, and the mice were euthanized and dissected. Biochemical parameters of liver function (alanine aminotransferase (ALT) and aspartate aminotransferase (AST)) and renal function (BUN and CRE) were measured from the collected blood by a fully automated biochemical analyzer (Rayto Chemray 240, China). Major organs (e.g., heart, kidney, lung, liver, and spleen) were collected for hematoxylin and eosin (H&E) staining.

### 2.13 Statistical analysis

All data were expressed as mean ± SD. Data analysis was calculated using GraphPad Prism 8 software. Data were compared using an unpaired two-tailed Student’s t-test between the two groups. More than two groups were compared using one-way ANOVA and Tukey’s multiple comparison test. A *p*-value < 0.05 was considered statistically significant (*p < 0.05, **p < 0.01, ***p < 0.001, ****p < 0.0001).

## 3 Results and discussion

### 3.1 Preparation and characterization of SA-PLGA@antagomiR-7

**FIGURE 1:**
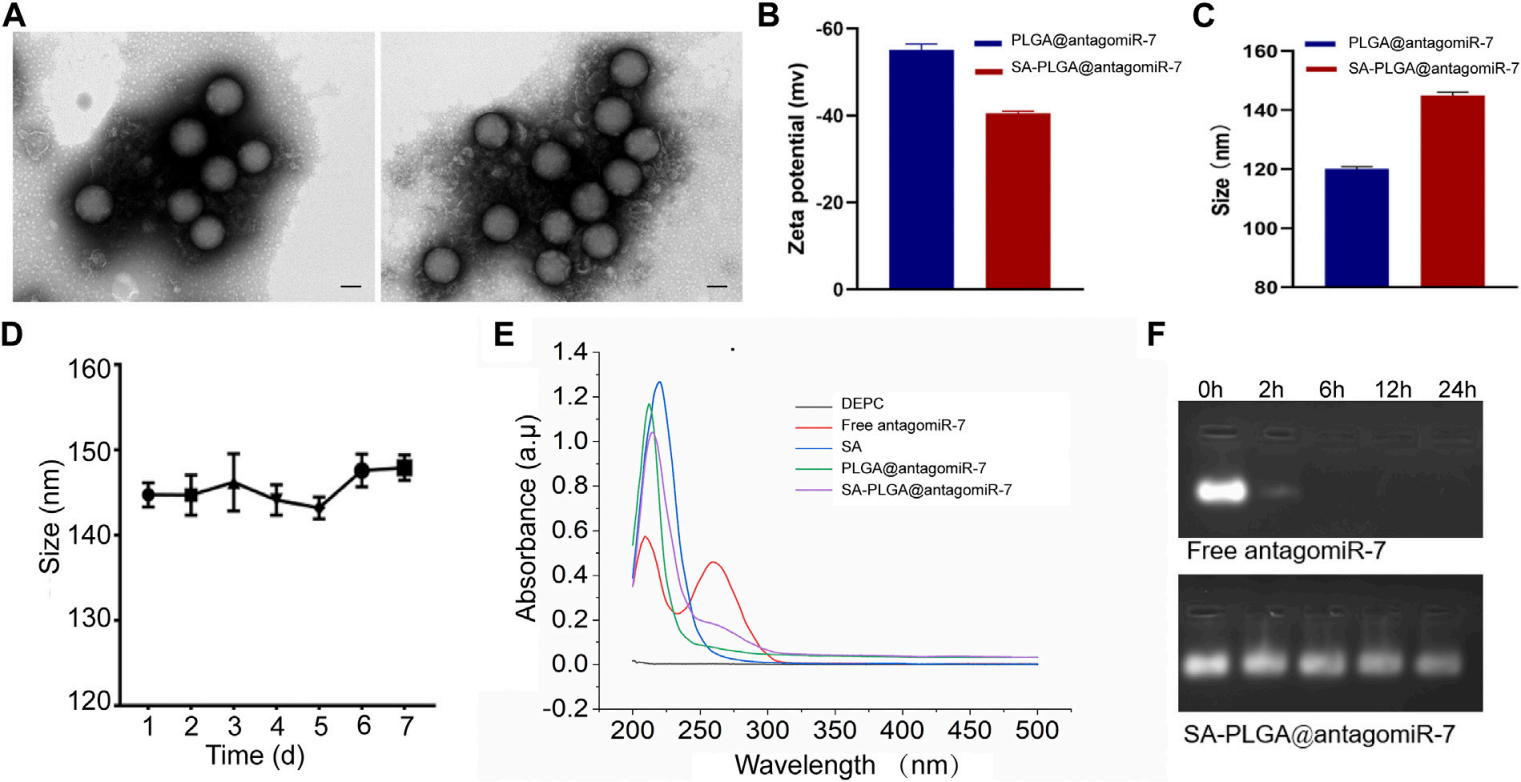
**(A)** TEM images of PLGA@antagomiR-7 (Left) and SA-PLGA@antagomiR-7 (Right). (Scale bar, 100 nm) **(B)** Zeta potential and **(C)** hydrodynamic diameter of PLGA@antagomiR-7 and SA-PLGA@antagomiR-7. **(D)** The particle size of SA-PLGA@antagomiR-7 within 1 week. **(E)** UV–vis absorption spectra. **(F)** Free antagomiR-7 or SA-PLGA@antagomiR-7 was incubated with RNAase A at different time points, and then the miRNA level in indicated groups was examined by electrophoresis on an agarose gel.

SA-PLGA@antagomiR-7 NPs were prepared by the double emulsification method. PLGA@antagomiR-7 and SA-PLGA@antagomiR-7 were stained with uranyl acetate and visualized by transmission electron microscopy (TEM), and the images obtained showed the spherical shape of the nanoparticles (Figure 1A). Meanwhile, the negative surface zeta potential of the nanoparticles decreased from −55.13 ± 0.77 mV to −40.55 ± 0.29 mV (Figure 1B). The hydrodynamic size of the nanoparticles increased from 120.1 ± 0.43 nm to 144.9 ± 0.66 nm (Figure 1C) after loading the ligand. Importantly, when SA-PLGA@antagomiR-7 was suspended in 1 × PBS, respectively, the nanoparticle size remained out of stable particle size for 7 days (Figure 1D), indicating its good size stability. The loading spectroscopy of miRNA was verified by UV-Vis absorption. As shown in Figure 1E, the absorption peaks of SA-PLGA@antagomiR-7 and PLGA@antagomiR-7 were consistent with antagomiR-7, indicating that antagomiR-7 was successfully encapsulated into the NPs. In addition, the *in vitro* release performance of SA-PLGA@antagomiR-7 was investigated at 37°С (Supplementary Figure S1). The release was relatively fast initially, reaching a cumulative release of 41.4% after 12 h. After that, the miRNA was slowly released, and the cumulative release of miRNA was 79.9% within 120 h. The results suggest that SA-PLGA@antagomiR-7 has controlled release properties. Then, we incubated free antagomiR-7 or SA-PLGA@antagomiR-7 with RNAase A at 37°C. The bands of free antagomiR-7 disappeared after 6 h. In comparison, visible bands were still present in the NPs group after 24 h, indicating that NPs could protect miRNA from RNase degradation (Figure 1F). In addition, the polydispersity index (PDI) of PLGA@antagomiR-7 NPs was 0.039 ± 0.0020, and the PDI of SA-PLGA@antagomiR-7 was 0.035 ± 0.0024, both of which were qualified samples (Supplementary Figure S2).

### 3.2 Cellular uptake and cytotoxicity of cells

**FIGURE 2:**
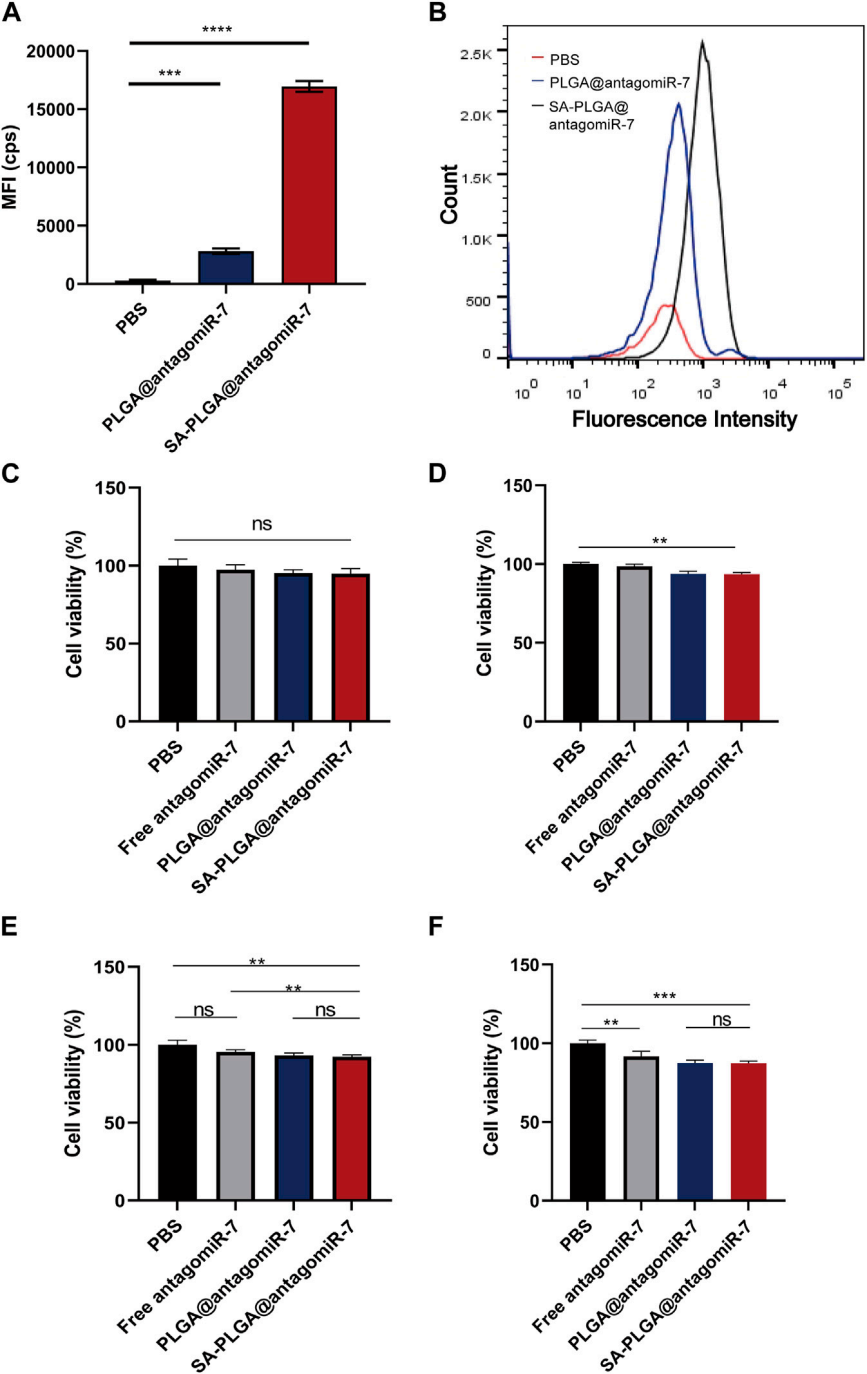
**(A)**The mean fluorescence intensity of B cell phagocytosis in lupus mice was determined by flow cytometric analysis after 6 h co-culture with Rho-PLGA@antagomiR-7 or Rho-SA-PLGA@antagomiR-7. **(B)** Flow histogram of **(A)**. B cell viability incubated with 100 μg/mL **(C)**, 200 μg/mL **(D)**, 400 μg/mL **(E)**, and 800 μg/mL **(F)** concentrations of PBS, antagomiR-7, PLGA@antagomiR-7, or SA-PLGA@antagomiR-7 for 24 h.

This study investigated whether SA-PLGA@antagomiR-7 NP could effectively deliver miRNA to splenic B cells. Cellular uptake of these B-cell NPs was investigated by labelling PLGA@antagomiR-7 and SA-PLGA@antagomiR-7 with Rhodamine (Rho). Cellular uptake was measured after 6 h of co-culture. According to flow cytometry data, the mean fluorescence intensity (MFI) of B cells treated with SA-PLGA@antagomiR-7 after 6 h of co-culture was much stronger than that after treatment with PLGA@antagomiR-7 (Figures 2A, B). This indicates that B cells took up more SA-PLGA@antagomiR-7 compared to PLGA@antagomiR-7. These results suggest that SA promotes PLGA@antagomiR-7 internalization through B cells.

We were used in this study to investigate whether PBS, Free antagomiR-7, PLGA@antagomiR-7, and SA-PLGA@antagomiR-7 are toxic to B cells. B cell viability was measured by CCK8 assay after 24 h of incubation with PBS, Free antagomiR-7, PLGA@antagomiR-7, and SA-PLGA@antagomiR-7. After 24 h of co-incubation, B cell viability in the SA-PLGA@antagomiR-7-treated group was not significantly different from that in the PBS group, remaining at 94.8% and 93.2% even at 100 μg/mL and 200 μg/mL concentration (Figures 2C, D). In contrast, at a concentration of 400 μg/mL and 800 μg/mL, B-cell viability was maintained at 92.3% and 90.1% in the SA-PLGA@antagomiR-7-treated group.(Figures 2E, F). These results indicate that SA-PLGA@antagomiR-7 is non-toxic and can be used as a biocompatible nanocarrier.

### 3.3 Fluorescence imaging and biodistribution of NPs in mouse

**FIGURE 3:**
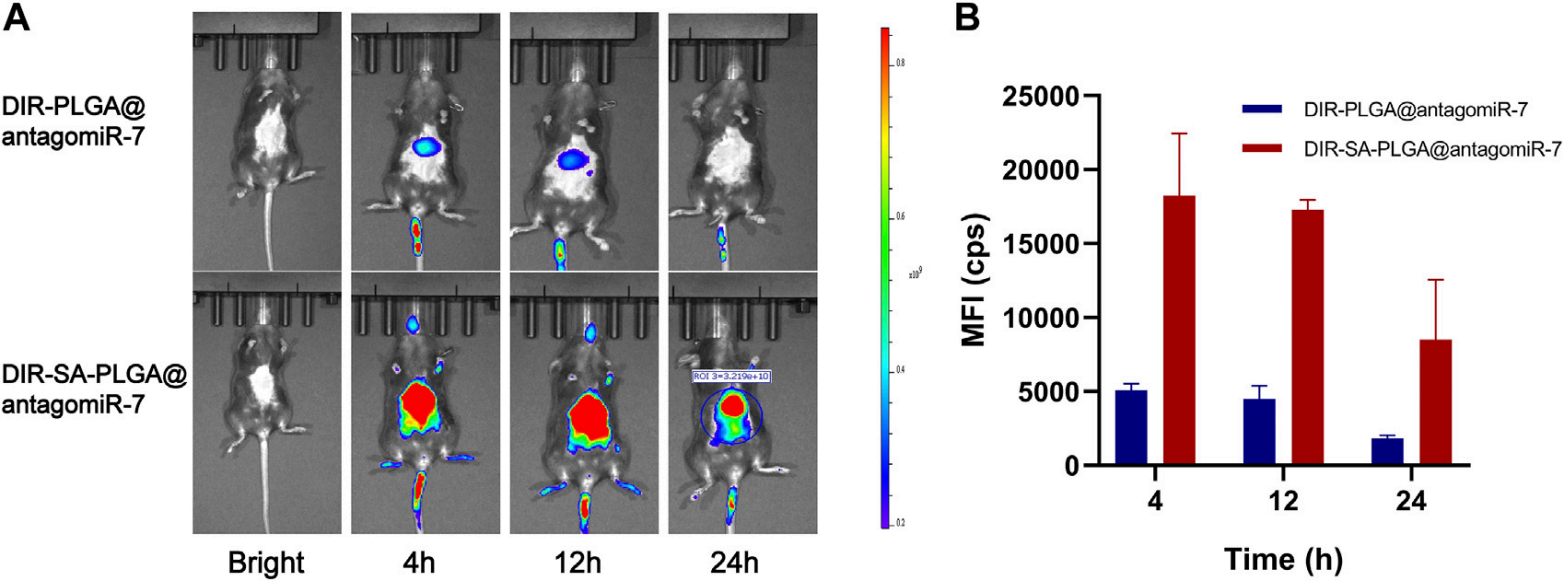
**(A)** *In vivo* fluorescence images of C57BL/6 mice at different times after intravenous injection of DIR-PLGA@antagomiR-7 or DIR-SA-PLGA@antagomiR-7. **(B)** Semi-quantitative analysis of fluorescence images of C57BL/6 mice (n = 3).

The spleen and liver isolate most of the administered nanoparticles, thus preventing them from entering the diseased tissue (Bronte and Pittet, 2013). Therefore, they constitute a significant barrier to using nanomedicines to treat certain diseases (e.g., tumours, Etc.). However, this represents an excellent opportunity to target splenocytes to treat SLE. We measured the biodistribution of SA-PLGA@antagomiR-7 *in vivo* to explore whether SA-PLGA@antagomiR-7 could preferentially enrich the spleen. Mice were injected with free DIR-SA-PLGA@antagomiR-7 or DIR-PLGA@antagomiR-7, and the fluorescence distribution was observed by fluorescence imager. After 12 h of injection, all mice showed strong fluorescence. However, at 24 h, the fluorescence signal was significantly diminished in the DIR-PLGA@antagomiR-7 group. In contrast, the abdomen of mice in the DIR-SA-PLGA@antagomiR-7 group still maintained a strong fluorescence signal (Figures 3A, B). These findings imply that SA-PLGA@antagomiR-7 can significantly enhance the retention of miRNA in the spleen and thus effectively deliver miRNA to splenic B cells for therapeutic purposes *in vivo*.

### 3.4 SA-PLGA@antagomiR-7 increases RTEN expression and reduces pro-inflammatory cytokine production

**FIGURE 4:**
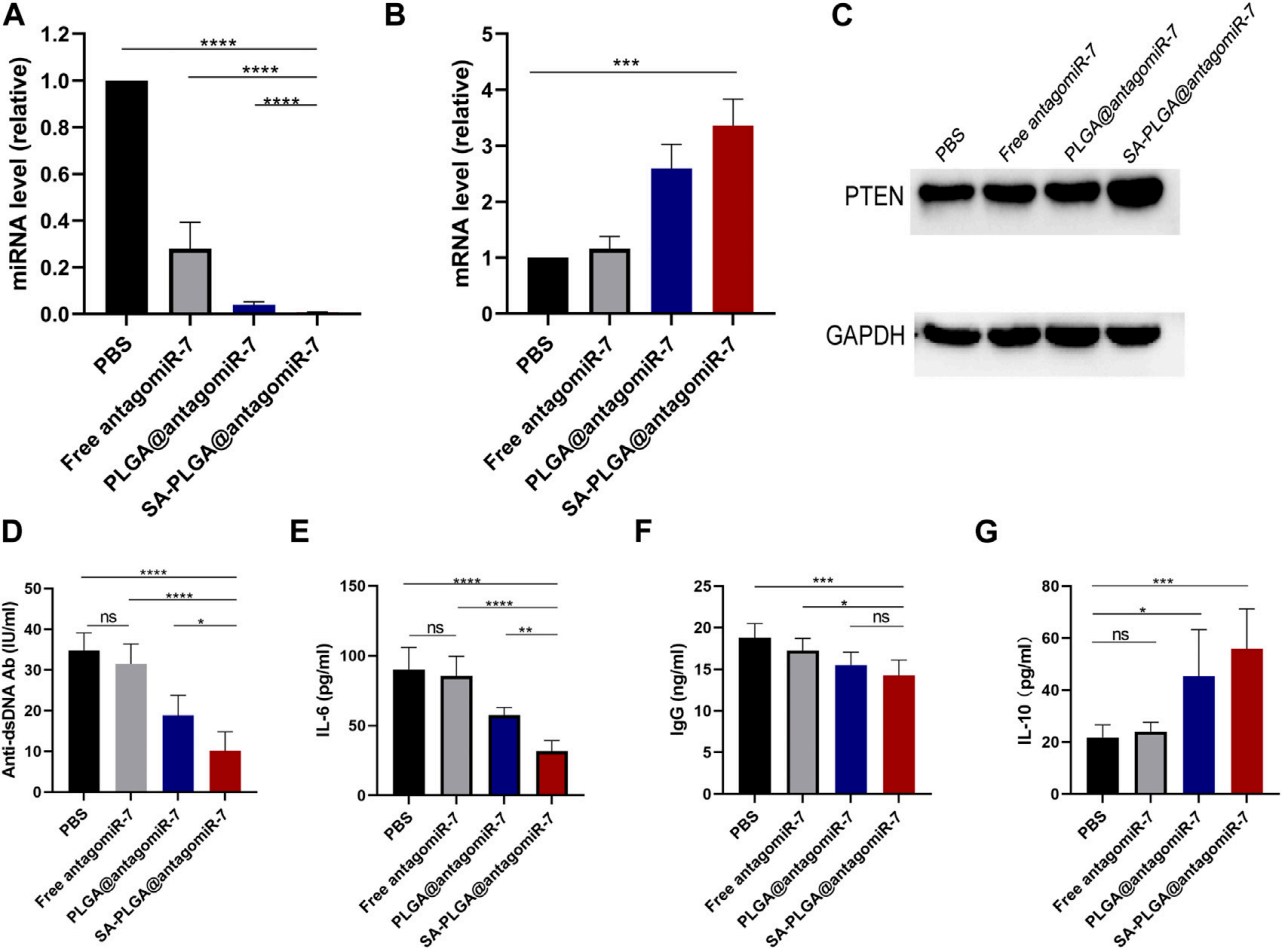
**(A)** The expression level of miR-7 in B cells of lupus mice treated with PBS, Free antagomiR-7, PLGA@antagomiR-7 and SA-PLGA@antagomiR-7 was determined by qRT-PCR. They were normalized according to U6 endogenous controls (*n* = 3). **(B)** The expression of PTEN mRNA in B cells was determined by RT qPCR. All PCR reactions were carried out at least three times. **(C)** Expression levels of PTEN protein in B cells of lupus mice treated with PBS, Free antagomiR-7, PLGA@antagomiR-7, and SA-PLGA@antagomiR-7 as assessed by Western blot. Serum was collected after treatment. Serum levels of anti-dsDNA Ab **(D)**, IL-6 **(E)**, IgG **(F)**, and IL-10 **(G)** were determined by ELISA. *p < 0.05, **p < 0.01. ***p < 0.001, ****p < 0.0001.

A previous study found that miR-7 levels elevated and positively correlated with reduced PTEN expression and a high percentage of plasma cells in MRL/Ipr lupus mice (Wu et al., 2014). In addition, miR-7 blockade with miR-7 antagomir corrected the increase in B-cell differentiation to plasma cells, a biological process mediated by regulation of the PTEN/AKT signalling pathway, and inhibited lupus disease activity (Wang et al., 2020). Therefore, we hypothesized that delivery of antagomiR-7 to splenic B cells to stabilize B cell hyperreactivity would have a therapeutic effect on SLE. To further verify that SA-PLGA@antagomiR-7 NP can effectively deliver antagomiR-7 into activated B cells, the relative levels of miR-7 and PTEN mRNA were measured. We found a significant decrease in miR-7 levels in SA-PLGA@antagomiR-7 NPs-treated B cells compared to PBS-treated B cells, whereas the free antagomiR-7 group had no significant effect on miR-7 levels, and the downregulated levels of PTEN mRNA were significantly reversed (Figures 4A, B). Then, we evaluated the effect of SA-PLGA@antagomiR-7 on RTEN expression in B cells from lupus mice. Among all treatment groups, SA-PLGA@antagomiR-7 treatment showed the most significant improvement in RTEN protein expression levels as assessed by protein blotting (Figure 4C). Thus, SA-PLGA@antagomiR-7 reduces the abnormal increase of miR-7 in splenic B cells of MRL/Ipr mice, regulates PTEN expression.

Since anti-dsDNA autoantibodies have been widely used to help establish the diagnosis and monitor the progression of SLE. Moreover, the anti-dsDNA antibody (Anti-dsDNA Ab) levels and IgG were positively correlated with the progression of SLE (Pisetsky, 2016). We next investigated whether SA-PLGA@antagomiR-7 could reduce autoantibody production *in vivo* and suppress the inflammatory response in lupus mice. At the end of treatment, serum levels of anti-dsDNA autoantibodies, IgG, IL-6, and IL-10 were measured by ELISA. Before starting treatment, we ensured baseline levels of laboratory markers including Anti-dsDNA autoantibodies, IgG, IL-6, and IL-10 in MRL/MpJ and MRL/Ipr mice (Supplementary Figures S3–S6). The data showed that serum anti-dsDNA autoantibodies (Figure 4D), IL-6 (Figure 4E) and IgG (Figure 4F) were significantly downregulated in the NPs group compared to PBS. At the same time, the anti-inflammatory cytokine IL-10 was elevated (Figure 4G). Furthermore, in our experimental setting, the treatment effect in the same dose of the free antagomiR-7 group was not observed in the NPs group as described above. These results suggest that the SA-PLGA@antagomiR-7 nano-delivery system dramatically improves the *in vivo* therapeutic efficiency of antagomiR-7, which implies that lupus mice treated with SA-PLGA@antagomiR-7 exhibit reduced production of anti-dsDNA Ab, and reduced inflammatory cytokines, and that SA-PLGA@antagomiR-7, a rationally designed nanocarrier, significantly improves inflammation levels. In addition, in our experimental setting, the treatment effect was not observed in the same dose of the free antagomiR-7 group as in the NPs group described above. These results suggest that the SA-PLGA@antagomiR-7 nano-delivery system dramatically improves the therapeutic efficiency of antagomiR-7.

### 3.5 SA-PLGA@antagomiR-7 treatment attenuates SLE progression

**FIGURE 5:**
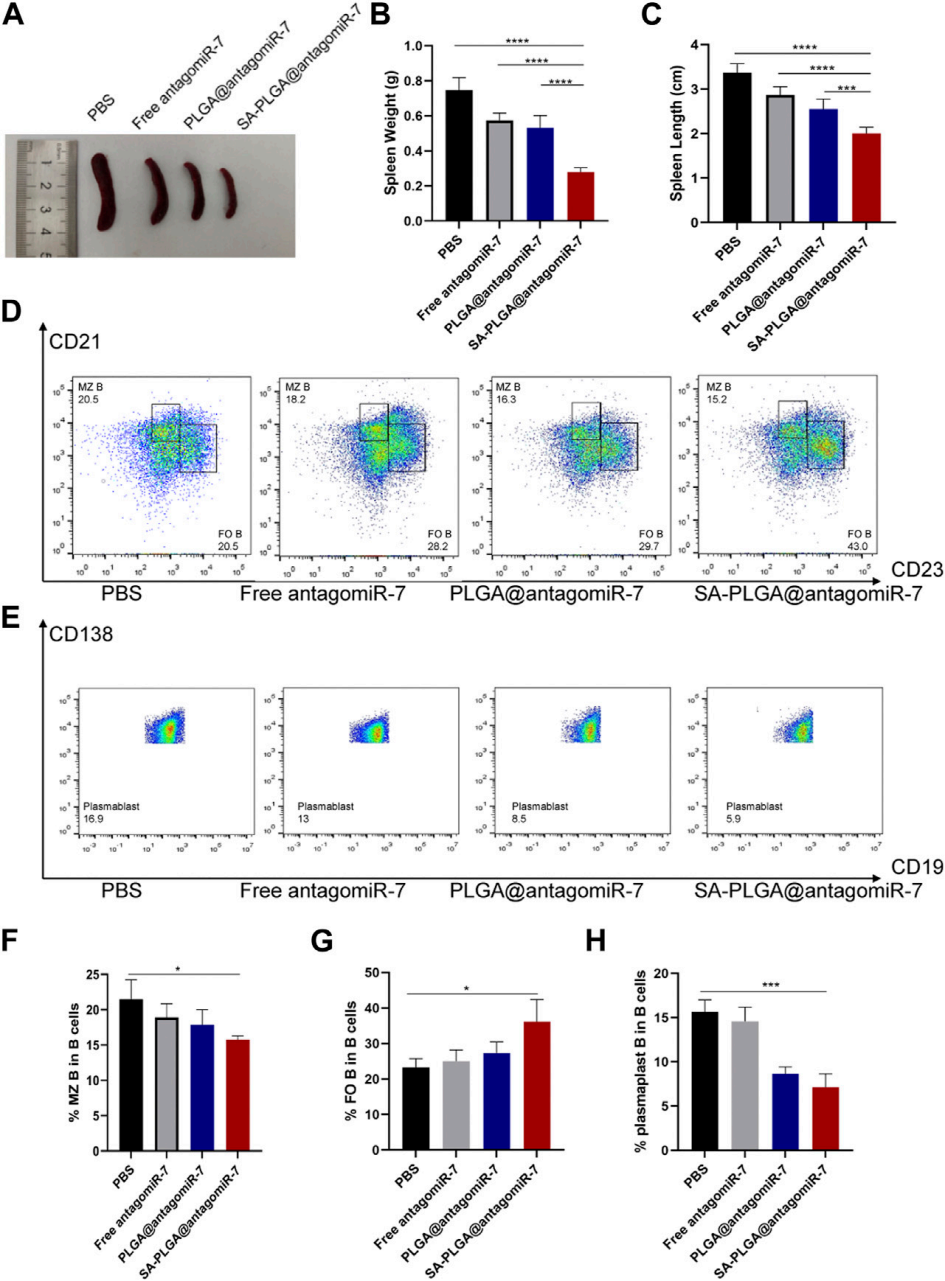
**(A)** Representative spleen images from SLE mice treated with PBS, Free antagomiR-7, PLGA@antagomiR-7, and SA-PLGA@antagomiR-7. **(B)** Spleen weight and length **(C)** of mice in different treatment groups. **(D)** Flow cytometry analyzed MZ B (CD21^high^CD23^low^) and FO B (CD21^mid^CD23^high^) cell percentages. **(E)** The percentage of plasmablast/plasma (CD19^−^CD138^+^) was determined by flow cytometry. The percentages of MZ B **(F)**, FO B **(G)** and plasmablast **(H)** cells were analyzed by flow cytometry.

Although some chemically modified miRNAs (e.g., Agomir or Antagomir) exert therapeutic effects on animal models by systemic administration, the dose of miRNAs is usually high, and the efficiency is relatively low. Encapsulation of miRNAs with appropriate nanocarriers protects them from nuclease degradation, prolongs circulation time, and reduces immune responses (Liu et al., 2015; Fu et al., 2018). In this study, we evaluated the therapeutic efficacy of SA-PLGA@antagomiR-7 in MRL/Ipr mice. In MRL/Ipr mice, abnormally proliferating B cells resulted in an abnormally enlarged spleen (splenomegaly), distinct from normal mice’s spleen. Our results showed that spleen weight and length were significantly lower in SA-PLGA@antagomiR-7 treated mice than in the PBS and free antagomiR-7 groups (Figures 5A–C). SA-PLGA@antagomiR-7 significantly reduced the weight of lymph nodes compared to the PBS and free antagomiR-7 groups (Supplementary Figure S7).

In addition, SA-PLGA@antagomiR-7 treatment significantly reduced the frequency of plasma cells, which led to a decrease in plasma cell production (Figures 5E, H). Correction of B-cell abnormalities and restricted GC formation, plasma cell differentiation and autoantibody production with SA-PLGA@antagomiR-7. With the development of lupus, increased populations of auto-reactive marginal zone B cells (MZ B) have been reported in several murine models, and their appearance positively correlates with pathological anti-dsDNA production (Sanz and Lee, 2010). By flow cytometric analysis of B-cell subpopulations, we found that the proportion of CD21^high^ CD23^low^ MZ B cells was increased in MRL/Ipr mice (Figures 5D, F) and decreased after treatment with SA-PLGA@antagomiR-7. On the other hand, CD21^mid^ CD23^high^ follicular B cells (FO B) were significantly reduced in the spleen of MRL/Ipr mice, but the number of FO B was significantly increased after SA-PLGA@antagomiR-7 treatment (Figure 5G). PTEN deficiency leads to a significant increase in MZ B cells and a corresponding decrease in FO B cells, affecting B-cell homeostasis. In contrast, SA-PLGA@antagomiR-7 effectively reverses B-cell abnormalities and normalizes splenic B-cell subtypes.

### 3.6 SA-PLGA@antagomiR-7 treatment reduces lupus nephritis (LN)

**FIGURE 6:**
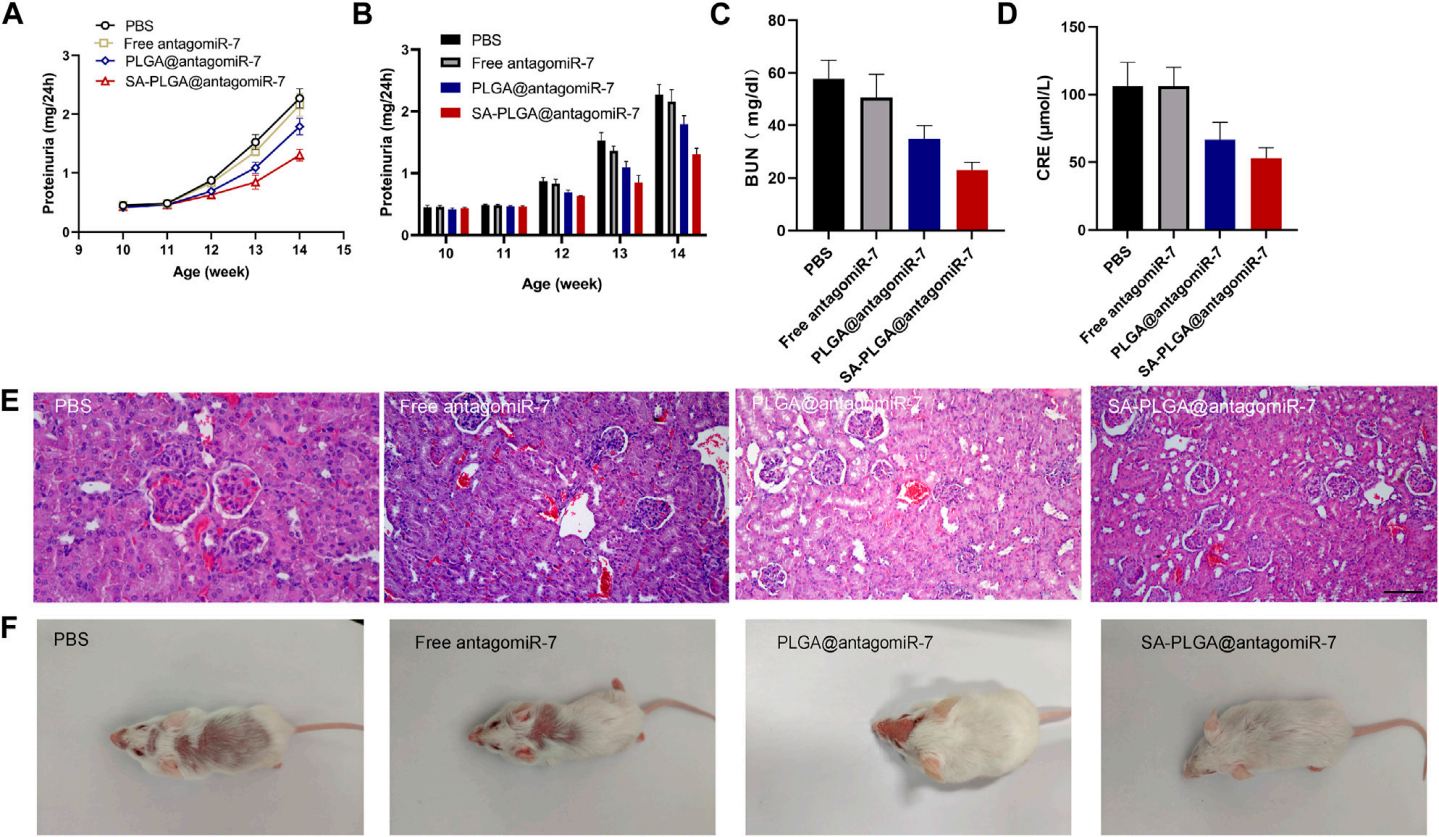
**(A)** Proteinuria was measured at different time points and statistic analysis **(B)**. **(C)** Serum BUN levels in SLE mice treated with PBS, Free antagomiR-7, PLGA@antagomiR-7, and SA-PLGA@antagomiR-7. **(D)** Serum creatinine levels in SLE mice treated with PBS, Free antagomiR-7, PLGA@antagomiR-7, and SA-PLGA@antagomiR-7. **(E)** H&E staining images of kidney sections from SLE mice treated with PBS, Free antagomiR-7, PLGA@antagomiR-7, and SA-PLGA@antagomiR-7. Scale = 100 μm. The scale bar in the last image can be applied to other images. **(F)** Skin lesions in SLE mice treated with PBS, Free antagomiR-7, PLGA@antagomiR-7, and SA-PLGA@antagomiR-7.

As lupus progresses, approximately 50% of SLE patients develop symptoms of lupus nephritis, and pathological examinations show that LN occurs in almost all SLE patients (Yap and Lai, 2015; Davidson, 2016). Lupus-related kidney injury remains one of the significant factors limiting the improvement of survival in patients with this disease (Zabaleta-Lanz et al., 2003; Hanly et al., 2016). Therefore, the effect of SA-PLGA@antagomiR-7 on reducing renal damage may reflect its therapeutic effect in SLE. Proteinuria, serum creatinine and urea nitrogen are essential indicators of disease progression. Proteinuria levels in lupus mice were monitored weekly. SA-PLGA@antagomiR-7 alleviated the upregulation of proteinuria levels in SLE mice (Figures 6A, B). Urea nitrogen and serum creatinine levels were also reduced in the SA-PLGA@antagomiR-7 group in SLE mice at the end of treatment (Figures 6C, D). We observed a gradual decrease in 24-h urine protein levels in the 13-week-old and 14-week-old lupus groups after treatment with PLGA@antagomiR-7 and SA-PLGA@antagomiR-7.

Similarly, all groups of lupus mice treated with SA-PLGA@antagomiR-7 showed the most significant decrease in 24-h urinary protein excretion levels. To further test the therapeutic efficacy of SA-PLGA@antagomiR-7 on LN, we performed a pathological examination of the kidneys of mice. The kidneys were cut into thin slices and stained with H&E; in addition, the histology of SA-PLGA@antagomiR-7-treated MRL/Ipr mice showed reduced glomerular enlargement in H&E staining (Figure 6E). Therefore, SA-PLGA@antagomiR-7 could more effectively mitigate the progression of kidney injury in lupus mice compared to Free antagomiR-7 and PLGA@antagomiR-7. In addition, SA-PLGA@antagomiR-7 treatment also significantly suppressed facial and back skin lesions, exhibiting an overall healthy appearance (Figure 6F).

### 3.7 Biocompatibility of SA-PLGA@antagomiR-7

**FIGURE 7:**
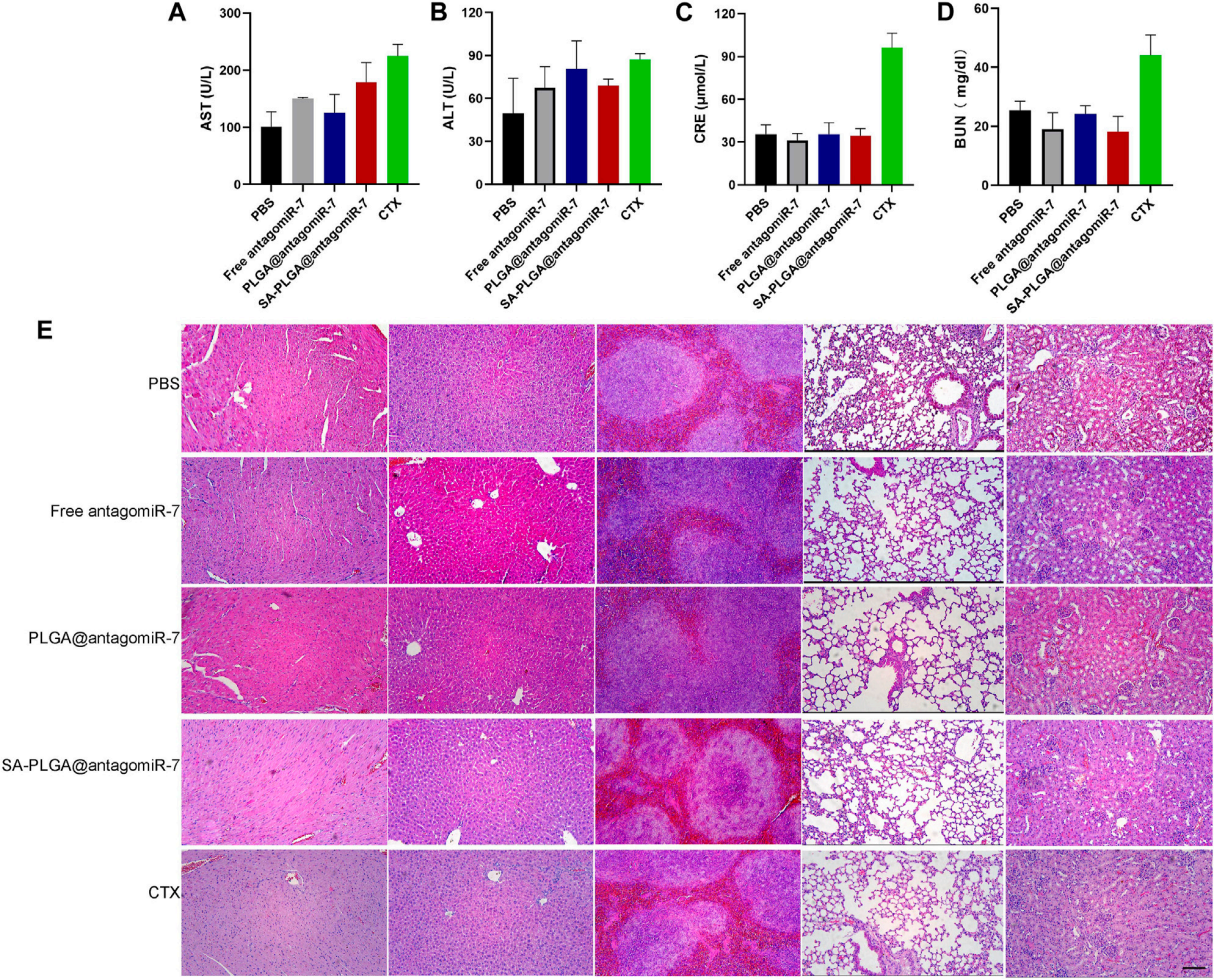
The liver and kidney function parameters of AST **(A)**, ALT **(B)**, CRE **(C)**, and BUN **(D)** in mice after 1 month of treatment were shown (*n* = 3). **(E)** Histopathological examination of vital, visceral organs of mice treated with PBS, Free antagomiR-7, PLGA@antagomiR-7, SA-PLGA@antagomiR-7, and CTX. Scale = 100 μm. The scale bar in the last image can be applied to other images.

As a drug carrier, it must have good biocompatibility. In our previous study, the toxicity of PLGA material was demonstrated to be negligible. In this study, we used SA-PLGA@antagomiR-7 as a carrier to deliver miRNA into splenic B cells for the treatment of SLE. Therefore, we will investigate the safety of PLGA@antagomiR-7 for treatment *in vivo*. Cyclophosphamide (CTX) is a traditional drug for treating SLE, but long-term use has side effects such as nephrotoxicity, and we use cyclophosphamide as a positive control. C57BL/6 mice were treated with PBS, Free antagomiR-7, PLGA@antagomiR-7, SA-PLGA@antagomiR-7 or CTX by intravenous injection twice a week for 1 month. It is critical to determine whether prolonged NP treatment has side effects on vital organs because they also have a high accumulation of NP. We examined blood biochemical parameters and histopathology of the vital organs. Serum AST and ALT levels were within the normal range between the groups (Figures 7A, B). In contrast, creatinine and urea nitrogen levels were significantly elevated in the CTX group (Figures 7C, D). Histological examination by H&E staining did not show any significant histopathological damage to the heart, liver, spleen, lungs, or kidneys (Figure 7E). This indicates that SA-PLGA@antagomiR-7 has good biocompatibility and no significant toxicity to essential organs such as liver and kidney. PLGA is one of the biodegradable polymers that is most frequently employed, because its hydrolysis produces the metabolite monomers lactic acid and glycolic acid (Hua et al., 2021; Piperno et al., 2021; Zhao et al., 2021). The use of PLGA for drug administration or biomaterial applications is associated with limited systemic toxicity since these two monomers are endogenous and easily degraded by the body via the Krebs cycle (Danhier et al., 2012; Hua et al., 2021). Moreover, polyester-based nanocarriers showed lower immunogenicity and improved release profile, complete biodegradation through natural pathways without accumulation in tissues (Chereddy et al., 2014; Zhang et al., 2020; Zhang et al., 2023). In conclusion, this fully demonstrates the excellent biosafety of SA-PLGA@antagomiR-7.

## 4 Conclusion

In this study, we successfully constructed the SA-PLGA@antagomiR-7 nano-delivery system. This nanosystem has effectively slowed the progression of SLE in mice, helped reduce the production of anti-dsDNA autoantibodies, reduced systemic inflammation, and attenuated renal injury with a high biosafety profile. In addition, our data demonstrate that SA-PLGA@antagomiR-7 can preferentially enrich pathological spleens and efficiently deliver antagomiR-7 to pathogenic splenic B cells in SLE models. In contrast, the same dose of free antagomiR-7 did not exhibit therapeutic effects on SLE. These results suggest that the SA-PLGA@antagomiR-7 nano-delivery system dramatically improves the therapeutic efficiency of antagomiR-7 *in vivo*. Furthermore, our study showed that antagomiR-7 could regulate PTEN expression and modulate B-cell hyperresponsiveness. Overall, this drug delivery system offers a promising strategy for SLE treatment by addressing B-cell hyperreactivity and pathogenic autoantibody production in this difficult-to-treat disease.

## Data Availability

The raw data supporting the conclusion of this article will be made available by the authors, without undue reservation.
