# Supplementary material for: Delivery of AntagomiR-7 through polymer nanoparticles for assisting B Cell to alleviate systemic lupus erythematosus
Source: Front Bioeng Biotechnol. 2023 Apr 19;11:1180302. doi: 10.3389/fbioe.2023.1180302 (PMC10154577; doi:10.3389/fbioe.2023.1180302)
Supplement: Supplementary file 1 [file DataSheet1.docx]

Supplementary Material

**Delivery of AntagomiR-7 through Polymer Nanoparticles for Assisting B Cell to Alleviate Systemic Lupus Erythematosus**

**Hui Guo^1†^, Jiangtao Ma^1,2†^, Yanli Zhang^1^, Yan Mao^1^, Ziwei Hu^4^, Ying Lin^4^, Feng Yu^4^, Wei Wang^1,2,3^* and Yaling Liu^1^***

1. Department of Dermatology, The Third Hospital of Hebei Medical University, Shijiazhuang, 050051, Hebei, People’s Republic of China

2. Orthopaedic Institution of Hebei Province, Hebei Orthopedic Clinical Research Center, The Third Hospital of Hebei Medical University, Shijiazhuang, 050051, Hebei, People’s Republic of China

3. School of Pharmaceutical Sciences, Southern Medical University, Guangzhou, 510515, Guangdong, People’s Republic of China

4. Institute of Otolaryngology, Head and Neck Surgery, Guangzhou Red Cross Hospital of Jinan University

† These authors have contributed equally to this work and share first authorship.

*** Correspondence:**Yaling Liu
yzling_liu1214@126.com

Wei Wang
wangwei199203@163.com

## Supplementary Figures


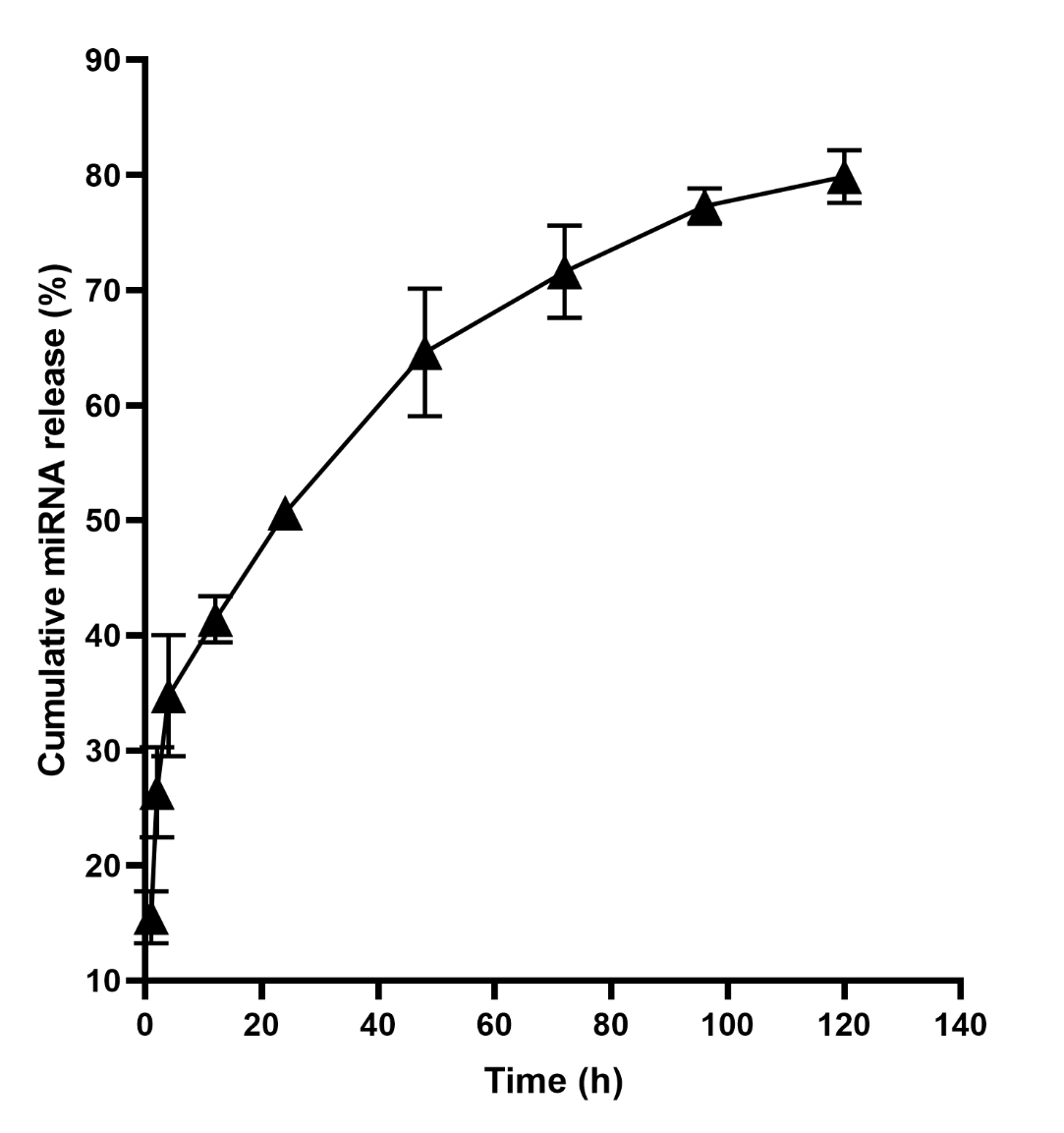


**Supplementary Figure S1 |** In vitro release profile of SA-PLGA@antagomiR-7.


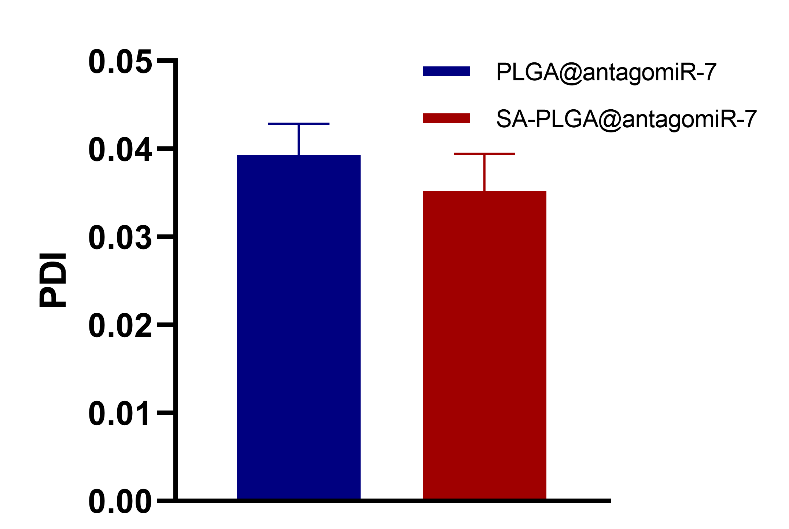


**Supplementary Figure S2 |** The PDI profile of SA-PLGA@antagomiR-7.


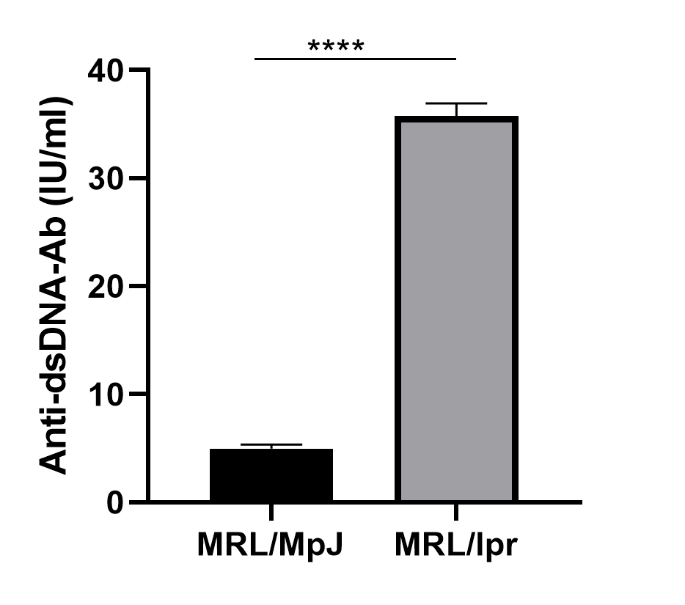


**Supplementary Figure S3 |** Serum levels of anti-dsDNA Ab of MRL/MpJ and MRL/Ipr mice were determined by ELISA.


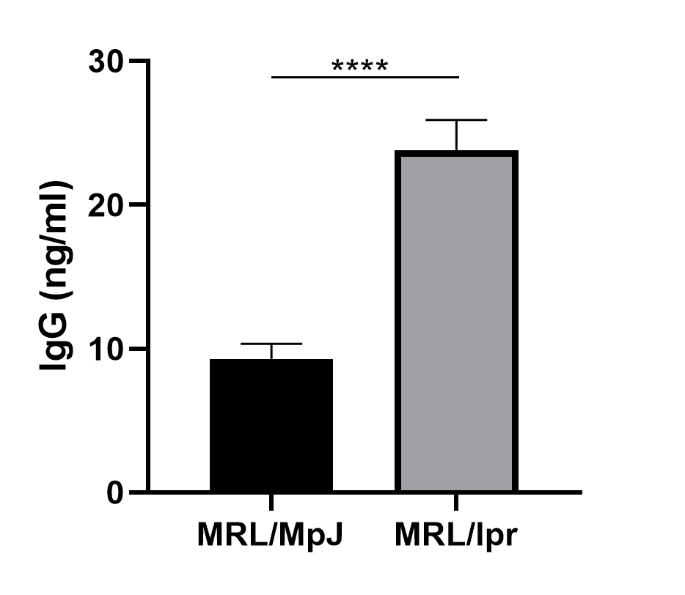


**Supplementary Figure S4 |** Serum levels of IgG of MRL/MpJ and MRL/Ipr mice were determined by ELISA.


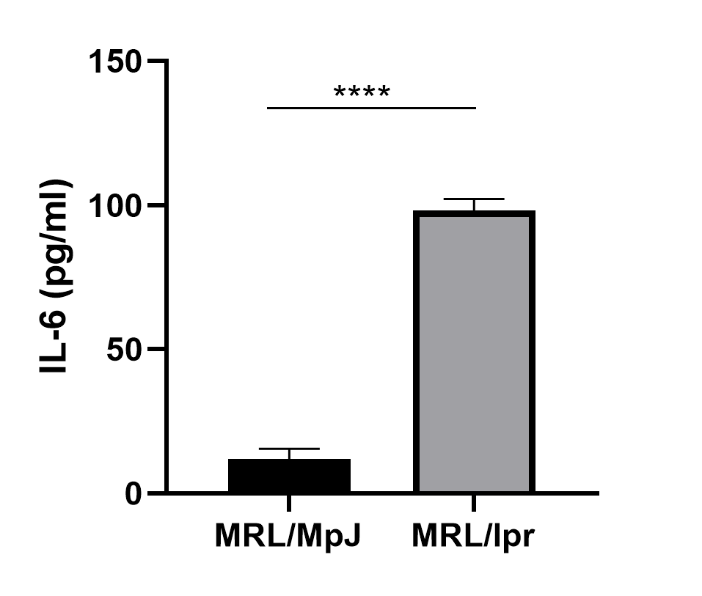


**Supplementary Figure S5 |** Serum levels of IL-6 of MRL/MpJ and MRL/Ipr mice were determined by ELISA.


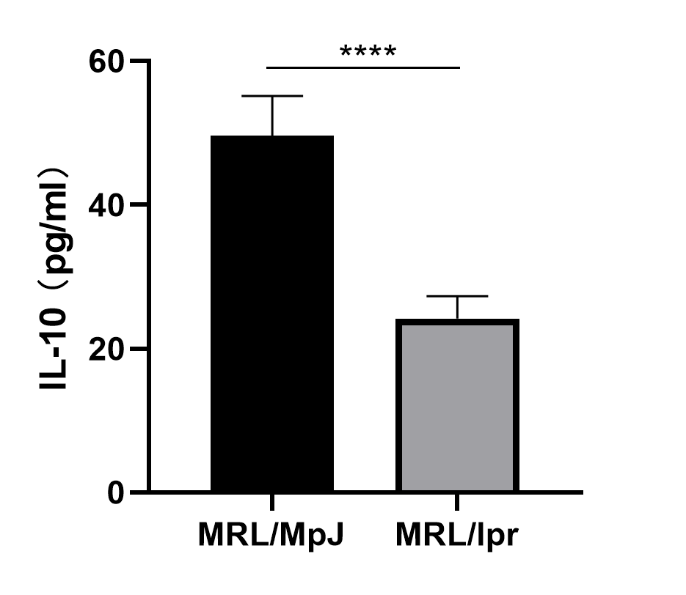


**Supplementary Figure S6 |** Serum levels of IL-10 of MRL/MpJ and MRL/Ipr mice were determined by ELISA.


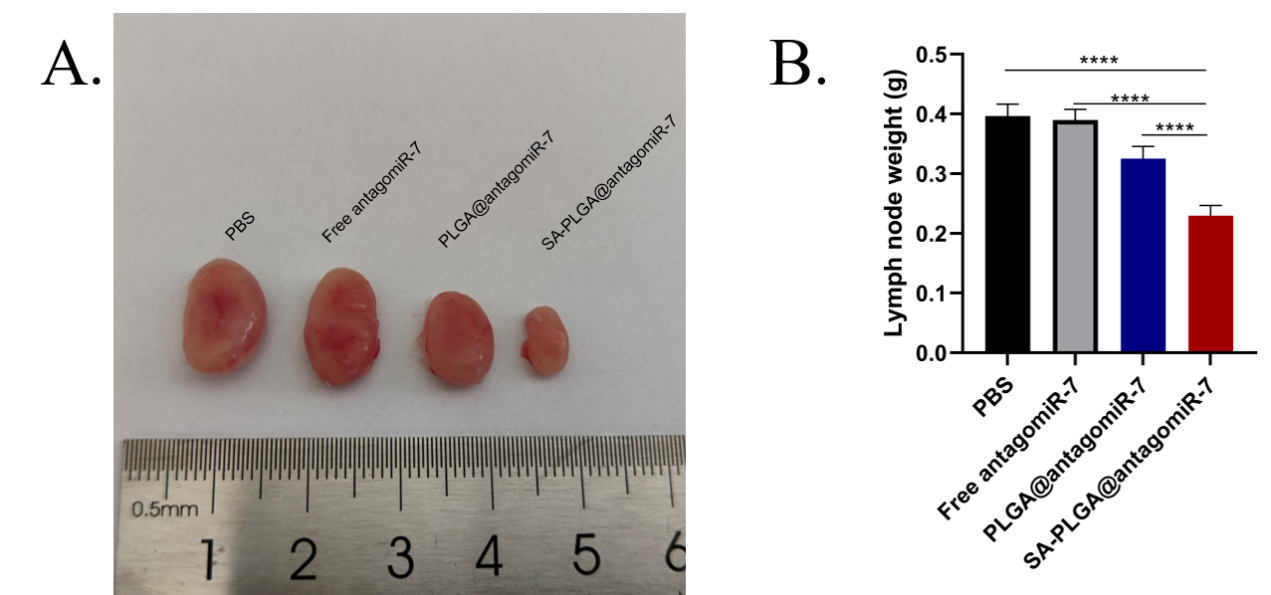


**Supplementary Figure S7 | (A)** Representative images of lymph nodes from PBS, Free antagomiR-7, PLGA@antagomiR-7, and SA-PLGA@antagomiR-7 treated MRL/Ipr mice. **(B)** Bar plot showing lymph nodes weight of different treatment groups as described in A. Results are expressed as mean ± SD (n=3).
